# GABA Production in *Lactococcus lactis* Is Enhanced by Arginine and Co-addition of Malate

**DOI:** 10.3389/fmicb.2016.01050

**Published:** 2016-07-06

**Authors:** Valérie Laroute, Chonthicha Yasaro, Waranya Narin, Roberto Mazzoli, Enrica Pessione, Muriel Cocaign-Bousquet, Pascal Loubière

**Affiliations:** ^1^Laboratoire d’Ingénierie des Systéms Biologiques et des Procédés, Université de Toulouse, Centre National de la Recherche Scientifique, Institut National de la Recherche Agronomique, Institut National des Sciences Appliquées, ToulouseFrance; ^2^Dipartimento di Scienze della Vita e Biologia dei Sistemi, Università degli Studi di TorinoTurin, Italy

**Keywords:** GABA, arginine, glutamate decarboxylase, ADI, malo-lactic fermentation

## Abstract

*Lactococcus lactis* NCDO 2118 was previously selected for its ability to decarboxylate glutamate to γ-aminobutyric acid (GABA), an interesting nutritional supplement able to improve mood and relaxation. Amino acid decarboxylation is generally considered as among the biochemical systems allowing lactic acid bacteria to counteracting acidic stress and obtaining metabolic energy. These strategies also include arginine deiminase pathway and malolactic fermentation but little is known about their possible interactions of with GABA production. In the present study, the effects of glutamate, arginine, and malate (i.e., the substrates of these acid-resistance pathways) on *L. lactis* NCDO 2118 growth and GABA production performances were analyzed. Both malate and arginine supplementation resulted in an efficient reduction of acidity and improvement of bacterial biomass compared to glutamate supplementation. Glutamate decarboxylation was limited to narrow environmental conditions (pH < 5.1) and physiological state (stationary phase). However, some conditions were able to improve GABA production or activate glutamate decarboxylation system even outside of this compass. Arginine clearly stimulated glutamate decarboxylation: the highest GABA production (8.6 mM) was observed in cultures supplemented with both arginine and glutamate. The simultaneous addition of arginine, malate, and glutamate enabled earlier GABA production (i.e., during exponential growth) at relatively high pH (6.5). As far as we know, no previous study has reported GABA production in such conditions. Although further studies are needed to understand the molecular basis of these phenomena, these results represent important keys suitable of application in GABA production processes.

## Introduction

**FIGURE 1:**
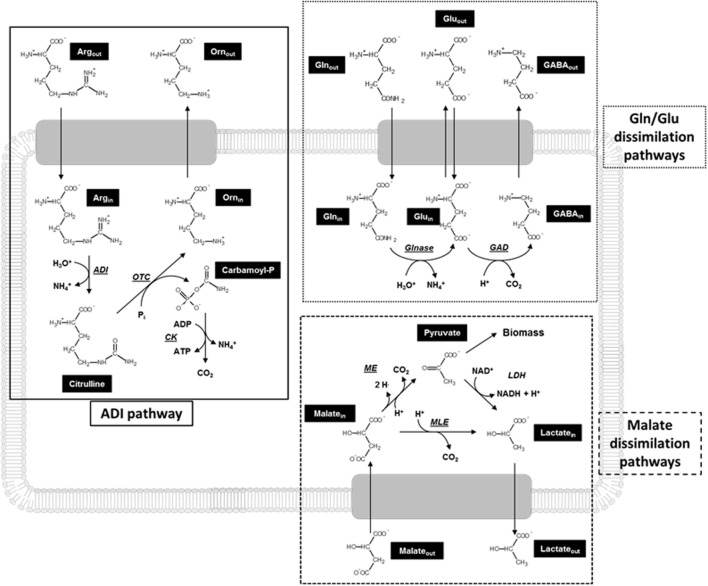
**Schematic representation of metabolic pathways potentially contributing to acid resistance in *Lactococcus lactis*.** *ADI*, arginine deiminase; Arg, arginine; *CK*, carbamate kinase; GABA, γ-aminobutyric acid; *GAD*, glutamate decarboxylase; Gln, glutamine; Glnase, glutaminase; Glu, glutamate; *ME*, malic enzyme; *MLE*, malolactic enzyme; Orn, ornithine; *OTC*, ornithine transcabamylase.

Lactic acid bacteria (LAB) are Gram-positive microaerophilic microorganisms extensively used in the agro-food industry because of their high lactic acid production and consequent food acidification. This is an appreciated feature for both prolongation of food shelf-life and biocontrol of food born infections, since most spoilage and pathogenic bacteria are acid-sensitive (Trias et al., 2008). The acid-resistance of LAB is based upon different, either constitutive or inducible, mechanisms which include: (i) cytoplasm alkalinization by H^+^ consumption through decarboxylation mechanisms, or arginine deiminase (ADI) pathway or urease reaction; (ii) changes in the composition of the cell envelope; (iii) production of general shock proteins (chaperones); and (iv) changes in cell density (for a review, see Cotter and Hill, 2003). Most of these strategies involve the expression of genes which improve cell resistance to adverse conditions. In lactococci the main metabolic mechanisms involved in pH homeostasis are ADI, malolactic fermentation (MLF) and glutamate decarboxylase (GAD) systems (**Figure 1**). Nowadays, little is known about possible interactions between these metabolic systems in *L. lactis*.

Arginine deiminase pathway consists of three reactions converting arginine to ornithine, NH_3_, CO_2_, and ATP. This route is catalyzed by three enzymes: ADI (converting arginine to citrulline and NH_3_), ornithine transcarbamylase (converting citrulline to ornithine and carbamoyl phosphate), and carbamate kinase (converting carbamoyl phosphate to NH_3_, CO_2_, and ATP), encoded by the genes *arcA*, *arcB*, and *arcC*, respectively (**Figure 1**). These enzymes appear to be acid resistant (Casiano-Colón and Marquis, 1988). The efficiency of the overall pathway is increased by arginine supplementation (Poolman et al., 1987). The ADI pathway provides both cytoplasm alkalinization, through release of NH_3_, and metabolic energy production, since ATP is generated by substrate level phosphorylation in the reaction catalyzed by carbamate kinase. The ADI systems of *Lactobacillus sakei* (Zuniga et al., 1998), *Enterococcus faecalis* (Simon et al., 1982), and LAB strains associated with cheese fermentations (Crow and Thomas, 1982) or colonizing oral cavity (Marquis et al., 1987; Dong et al., 2002) are subject to catabolite repression by glucose. However, in *L. sanfranciscensis* (De Angelis et al., 2002), *Oenococcus oeni*, and other wine LAB (Liu et al., 1996) glucose and arginine can be concomitantly catabolized.

Malolactic fermentation is the conversion of dicarboxylic malic acid to monocarboxylic L-lactic acid by malolactic enzyme (**Figure 1**). L-lactate is excreted via electrogenic transporters, i.e., by either lactate–malate antiporter (*L. lactis*) or lactate uniport (*O. oeni* and *L. plantarum*) (Konings et al., 1997) depending on the strain, which allow production of proton motive force. Furthermore, due to the p*K*_a_ difference of the carboxylic groups of malate and lactate, replacement of malate by lactate results in alkalinization of the extracellular medium. A further relevant malate dissimilation pathway which can contribute to acid resistance of LAB is oxidative decarboxylation of malate to pyruvic acid (catalyzed by malic enzyme; Landete et al., 2013). It is generally accepted that malate utilization through MLF cannot sustain growth *per se* (since lactate cannot be used as a carbon source by most LAB), while its metabolism via malic enzyme enables LAB to use it as the only carbon source. Distribution of malic enzyme currently seems restrained to fewer strains (including *L. lactis* NCDO 2118) with respect to MLF (Landete et al., 2013; Oliveira et al., 2014). However, malic enzyme has been studied at much lesser extent than MLF and, currently, contribution of malic enzyme pathway to LAB metabolism cannot be precisely evaluated.

Like other amino acid decarboxylations, glutamate conversion to γ-aminobutyric acid (GABA) is an important strategy to counteract excess of acidity (Schelp et al., 2001; Van de Guchte et al., 2002; Pessione, 2012) since the reaction itself is proton consuming and results in alkalinization of the cytoplasmic compartment (Small and Waterman, 1998). GAD system has been reported in both Gram-positive and Gram-negative bacteria (Small and Waterman, 1998; Bhagwat and Bhagwat, 2004; Cotter et al., 2005; Tramonti et al., 2006) and includes proton-consuming decarboxylation of glutamate by GAD in the cytoplasm and cell membrane-located electrogenic glutamate/GABA antiporters which generate proton motive force (Molenaar et al., 1993; Higuchi et al., 1997; Lu et al., 2013; Tsai et al., 2013) (**Figure 1**). The expression of GAD in *L. lactis* is increased by low pH and glutamate supplementation (Sanders et al., 1998). Recent studies have identified a further acid-resistance mechanism in *L. reuteri* and *Escherichia coli* which is based on glutamine and can be interpreted as an “extension” of the GAD system (Lu et al., 2013; Teixeira et al., 2014). Both glutaminase, which catalyzes glutamine deamidation (producing ammonia and glutamate), and GAD are present in the cytoplasm of these strains and contribute to intracellular alkalinization. These studies indicated that GadC, which had been previously identified as a glutamate/GABA antiporter, is also able to mediate uptake of glutamine or extrusion of glutamate. A gene encoding a putative glutamine/GABA antiporter has been identified in the genome of *O. oeni* PSU-1 also (Mills et al., 2005).

*Lactococcus lactis* NCDO 2118 is able to biosynthesize GABA by glutamate decarboxylation. A previous transcriptomic and proteomic study demonstrated that ADI pathway genes (*arcA, arcD1*, *arcB*, and *arcC2)* are down-regulated in glutamate-supplemented/GABA-producing conditions, thus suggesting that glutamate decarboxylation and arginine deimination are competing routes in this strain (Mazzoli et al., 2010). The present investigation aimed to better establish the relative contribution of GAD, ADI, and MLF pathways in energy metabolism and acid resistance of *L. lactis* NCDO 2118 and possible reciprocal interactions of these metabolic systems.

## Materials and Methods

### Bacterial Strain

*Lactococcus lactis* subsp. *lactis* NCDO 2118 from vegetable origin was used throughout this study. This strain was selected during preliminary studies as the only one able to biosynthesize detectable amounts of GABA among the *L. lactis* strains available in the laboratory microbial collection (LISBP of INSA-Toulouse, France).

### Culture Conditions

#### Cultures in Tubes

Cultures were grown in the chemically defined medium (CDM; Otto et al., 1983; Poolman and Konings, 1988), containing glucose (20 g L^-1^) under anaerobic conditions, i.e., in N_2_ atmosphere, in butyl rubber-stoppered tubes at 30°C. The initial pH was 6.6. Furthermore, different concentrations of glutamate and/or arginine and/or malate were added into the medium depending on the study. All the experiments were performed in duplicate. Inoculation was with cells from precultures harvested during the exponential phase and concentrated in order to obtain an initial optical density at 580 nm (OD_580_) of 0.05 in the tubes. During incubation, 1 mL samples were taken every 30 min so as to measure the OD_580_ with Spectronic 301 spectrophotometer (Milton Roy, Pont Saint Pierre, France). The maximum growth rate (μ_max_) was then determined. pH was also regularly measured with pH meter (Metrohm 744, Villebon Sur Yvette, France).

#### Cultures in Fermenter

Bacterial cultures were performed in duplicate in 2 L Biostat B plus fermenter (Sartorius, Melsungen, Germany) filled with glucose (20 g L^-1^) containing-CDM or the same medium supplemented with 5 g L^-1^ (34 mM) glutamate and/or 5 g L^-1^ (29 mM) arginine and/or 20 g L^-1^ (149 mM) malate. Cultures were incubated at 30°C in anaerobiosis, obtained by slight N_2_ overpressure. pH was maintained at 6.6 by KOH addition until cultures reached OD_580_ = 1, in order to reach enough biomass for further analytical procedures, and then pH was not regulated anymore. Bacterial growth was monitored by measurement of OD_580_ (Libra S11, Biochom, 1 Unit of absorbance is equivalent to 0.3 g L^-1^). Samples were collected every 30 min for HPLC determination of metabolite concentration in the growth medium.

### Metabolite Determination

Glucose, malate, and metabolite (i.e., lactate, acetate, formate and ethanol) concentrations were measured in culture super natants by high performance liquid chromatography (Agilent Technologies 1200 Series, Waldbronn, Germany) using a HPX87H^+^ Biorad column and the following conditions: a temperature of 48°C, eluent H_2_SO_4_ (5 mM) at a flow rate of 0.5 mL min^-1^, and dual detection (refractometer and UV).

Free amino acid and GABA concentration in culture super natants was measured by HPLC system (Agilent Technologies 1200 Series, Waldbronn, Germany). Prior to HPLC deter mination, proteins in the samples were precipitated by adding four volumes of methanol followed by overnight incubation on ice. The mixture was centrifuged and the supernatant kept for HPLC analysis. Amino acids were automatically derived with OrthoPhtalic Aldehyde (OPA) and 9-fluorenylmethyl-chloroformiate (FMOC-C1). The derivatives were separated on Hypersil AA-ODS column (Agilent Technologies) at 40°C by a linear gradient of acetate buffer (pH 7.2) with triethylamin (0.018 %), tetrahydrofuran (0.3%) and acetonitrile. A diode array detector, at 338 nm for OPA derivatives and 262 nm for FMOC derivatives, was used.

### Statistical Methods

Student’s *t*-test was applied to each parameter (**Table 1**) in order to detect significant differences between culture conditions (CDM or CDM supplemented with glutamate as the references). A *p*-value lower than 0.025 was considered as significant.

**Table 1:** Maximal specific rates; maximal biomass, time, and pH at the growth arrest; glucose consumption, lactate, and γ-aminobutyric acid (GABA) production, and pH at 48 h; during growth of *L. lactis* NCDO 2118 on seven different synthetic media.

Parameter	CDM	CDM^1^	CDM^1^	CDM^1^	CDM^2^	CDM^2^	CDM^2^
		+Glu	+Arg	+Malate	+Arg+Glu	+Glu+Malate	+Arg+Glu+Malate
**Maximal specific rates**							
μ_max_ (h^-1^)	0.97 ± 0.10	0.97 ± 0.04	1.01 ± 0.11	0.90 ± 0.02	0.89* ± 0.01	0.81* ± 0.01	0.85* ± 0.00
*q*_glucose_ (mmol g^-1^ h^-1^)	27.7 ± 3.4	26.9 ± 5.3	24.6 ± 2.1	26.6 ± 4.4	27.0 ± 3.6	32.0 ± 3.1	28.8 ± 7.9
ν_lactate_ (mmol g^-1^ h^-1^)	46.5 ± 3.0	49.9 ± 6.8	43.8 ± 3.6	42.1 ± 8.1	41.6* ± 4.8	56.2** ± 2.2	56.2 ± 13.9
*q*_malate_ (mmol g^-1^ h^-1^)				29.5 ± 0.2		24.8 ± 0.1	21.5 ± 2.4
*q*_glutamine_ (mmol g^-1^ h^-1^)	1.22 ± 0.27	0.72 ± 0.22	0.97 ± 0.25	1.69 ± 0.67	0.51 ± 0.14	1.39** ± 0.31	1.20** ± 0.15
*q*_arginine_ (mmol g^-1^ h^-1^)	1.44 ± 0.26	1.39 ± 0.58	9.80** ± 2.26	2.69** ± 0.44	7.91** ± 1.18	3.3** ± 0.35	14.58** ± 0.83
**At growth arrest**							
Biomass (g L^-1^)	0.98 ± 0.00	1.00 ± 0.11	1.96** ± 0.06	1.86* ± 0.17	1.98** ± 0.20	1.67** ± 0.02	2.20** ± 0.01
Time (h)	5	6	6	7	6	9	9
pH	4.94 ± 0.09	5.14 ± 0.18	5.07 ± 0.05	5.76* ± 0.13	5.31 ± 0.30	5.78** ± 0.06	6.22** ± 0.00
Glucose cons (mM)	34.4 ± 4.0	43.4 ± 5.9	70.7* ± 5.1	85.7** ± 5.0	63.4** ± 3.8	80.4** ± 0.9	71.1** ± 0.07
Lactate produced (mM)	65.6 ± 0.5	78.6 ± 8.4	123.3** ± 6.8	275.2* ± 4.7	112.5 ± 7.1	267.7** ± 6.9	228.05** ± 3.1
GABA (mM)	0	0.08 ± 0.01	0.32 ± 0.88	0.14 ± 0.61	0.17 ± 0.20	1.89** ± 0.14	1.71** ± 0.13
**At 48 h**							
Glucose cons. (mM)	51.31 ± 1.7	68.93 ± 17.9	89.9** ± 8.7	105.5** ± 0.7	89.4 ± 12.6	106.8** ± 3.7	121.2** ± 2.7
Lactate produced (mM)	102.2 ± 4.7	130.3 ± 24.8	153.3** ± 7.1	318.5** ± 10.6	166.2 ± 13.6	325.3** ± 8.0	353.3** ± 4.0
GABA (mM)	0.44 ± 0.02	3.12* ± 0.85	1.88** ± 0.25	0.38 ± 0.13	8.60* ± 2.0	2.54 ± 0.03	5.22 ± 0.33
pH	4.20 ± 0.02	4.52* ± 0.09	4.47** ± 0.03	4.95** ± 0.08	4.50 ± 0.21	4.90** ± 0.01	4.97** ± 0.00
Ornithine (mM)	0.42 ± 0.03	0.58 ± 0.37	34.90** ± 4.40	0.47 ± 0.18	36.95** ± 2.41	0.07 ± 0.01	25.81** ± 1.04
Citrulline (mM)	0	0	2.07** ± 0.46	0	1.14** ± 0.07	0	3.63** ± 0.23

ν_*lactate*_ is calculated only with lactate coming from glucose, and not from malate. Arg, arginine; Glu, glutamate. Values are means ± standard deviation. Parameters were compared to (1) CDM or (2) CDM+Glu condition with Student *t*-test. The statistical significance of the test was represented with (*) or (**) according to *p*-value threshold of 0.025 and 0.01, respectively.

## Results

### Growth and Metabolism of *L. lactis* NCDO 2118 in Control Conditions

**FIGURE 2:**
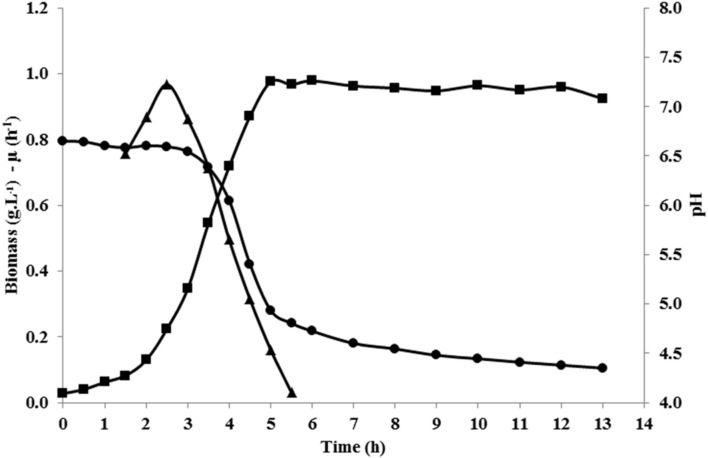
**Evolution of biomass (g L^-1^; ■), specific growth rate (h^-1^; ▲), and pH (●) during growth of *L. lactis* NCDO 2118 in chemically defined medium (CDM)**.

Fermentation profiles of *L. lactis* NCDO 2118 in glucose-containing chemically defined medium (CDM), in unregulated pH conditions, were determined. Growth started immediately after inoculum at maximal growth rate (μ_max_ = 0.97 h^-1^), and stopped after 5 h, at a biomass concentration of about 1 g L^-1^ (**Figure 2**; **Table 1**). At that time, the pH was about 4.9–5.0, and it further gradually decreased during the stationary phase until 4.2 (13 h after inoculum). *L. lactis* NCDO 2118 showed high maximal glucose consumption rate (27.7 mmol g^-1^ h^-1^) and exhibited homolactic metabolism all along the growth phases, leading to accumulation of 65.6 mM lactate (**Table 1**). Growth stopped before glucose depletion. We have performed cultures in the same medium but at regulated pH (6.6) during all the fermentation. In these conditions, growth continued until glucose exhaustion, demonstrating that acidic pH is responsible for the growth arrest in our control conditions.

The growth medium used in this study did contain no glutamate while low concentrations of arginine and glutamine (0.6 and 3.0 mM, respectively) were present. Arginine was quickly exhausted (*q*_max_ = 1.44 mmol g^-1^ h^-1^) and stoichiometrically converted to ornithine and citrulline. About half of the initial glutamine was consumed (*q*_max_ = 1.22 mmol g^-1^ h^-1^) in 48 h, leading to accumulation of 0.5 mM of glutamate and 0.4 mM of GABA in the growth medium (**Table 1**).

### Effect of Glutamate or Arginine or Malate Supplementation on Growth and Metabolism of *L. lactis* NCDO 2118

In order to study the effect of glutamate or arginine or malate on metabolic profiles of *L. lactis* NCDO 2118 cultures were performed in glucose-CDM medium supplemented with each of these single compounds at regulated and unregulated pH as described below. GABA was never produced in regulated pH conditions (pH = 6.6). It was detected at unregulated pH and only cultures performed in these conditions are described below.

#### Effect of Glutamate

**FIGURE 3:**
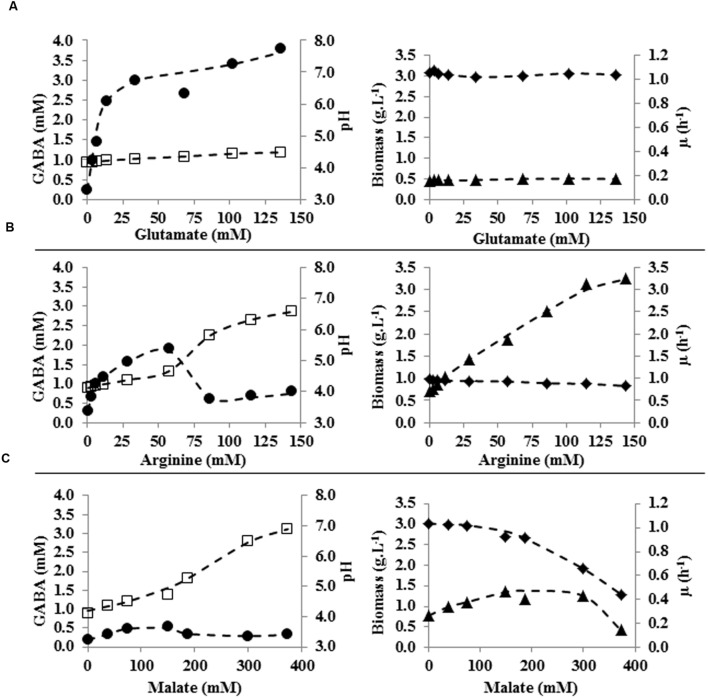
**Specific growth rate (h^-1^; ◆) of *L. lactis* subsp. *lactis* NCDO 2118, biomass (g L^-1^; ▲), pH (□), and GABA production (mM; ●) in CDM containing various concentrations of glutamate **(A)**, arginine **(B)**, or malate **(C)** at 48 h of culture. (---) trend curve**.

**FIGURE 4:**
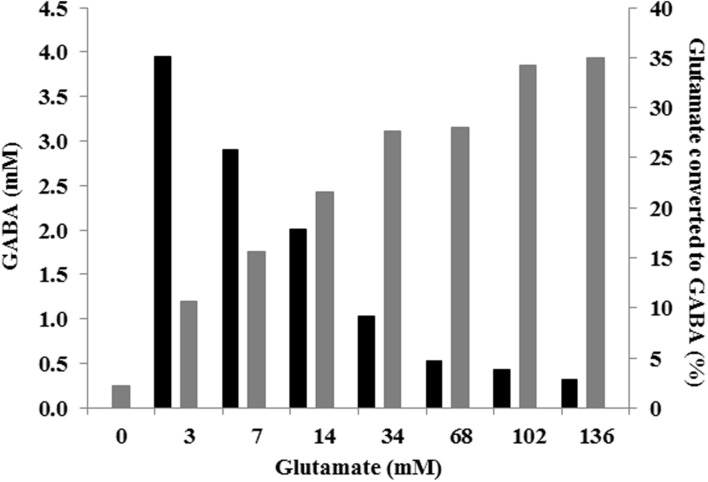
**GABA production (mM; gray) and percentage of glutamate converted to GABA (black) according to various initial glutamate concentrations in CDM, at 48 h of culture**.

The effect of different glutamate concentrations, ranging from 0 to 20 g L^-1^ (0–136 mM) was tested in tube cultures. Neither specific growth rate nor final biomass was affected by the different glutamate concentrations used (**Figure 3A**). However, slight variations in final pH and, more importantly, changes in amounts of accumulated GABA were observed among the different glutamate-supplemented cultures. The higher was glutamate supplementation, the higher was the final pH and the amount of GABA which was accumulated (pH = 4.1, 0.3 mM of GABA in cultures without glutamate supplementation; pH = 4.5, 3.8 mM GABA in cultures supplemented with 136 mM glutamate). It is worth noting that glutamate/GABA conversion yield was not the same in each condition since it was higher in cultures supplemented with lower glutamate concentration (i.e., 35 % in cultures containing 3.4 mM glutamate) and progressively decreased at higher glutamate supplementation (i.e., 3% in cultures containing 136 mM glutamate; **Figure 4**).

The medium containing 5 g L^-1^ glutamate (34 mM) retained our attention since it displayed high production of GABA with intermediary glutamate/GABA conversion yield. This condition was also previously used for transcriptome–proteome analysis (Mazzoli et al., 2010). Cultures were performed in fermenter in order to provide detailed metabolic parameters. As determined for tube-cultures (see above), glutamate supplementation did not affect μ_max_ and final biomass with respect to control cultures. Glutamate supplementation did not have any effect on glucose consumption rate, although a slightly higher glucose amount was consumed in these condition leading to accumulation of higher amounts of lactic acid (**Table 1**). Both final pH and GABA accumulation at 48 h were higher in glutamate-supplemented culture, thus confirming results obtained in tube cultures. It is worth noting that the amount of glutamine consumed was similar to control cultures, although maximal glutamine consumption rate was significantly lower, and that 5.5 mM of glutamate was consumed leading to accumulation of 3.12 mM GABA (**Table 1**). Finally, glutamate supplementation did not display any significant influence on arginine consumption rate (**Table 1**).

#### Effect of Arginine

Cultures of *L. lactis* NCDO 2118 were performed in tubes containing glucose-CDM medium supplemented with arginine concentrations ranging from 0 to 25 g L^-1^ (0–144 mM). Increasing amounts of supplemented arginine progressively caused: (i) a slight decrease of the maximal growth rate (from 1 to 0.8 h^-1^); (ii) a strong increase of both the final biomass, and (iii) final pH (from pH = 4.1 to pH = 6.6; **Figure 3B**). Arginine was depleted 6 h after inoculum in every tested culture and progressive alkalinization of final pH (proportional to increasing initial concentration of arginine) was observed. This was likely related to production of higher amounts of NH_3_ by arginine metabolization through the ADI route. Interestingly, arginine addition up to 10 g L^-1^ (57 mM) progressively enhanced GABA production up to 1.9 mM (**Figure 3B**). Since the medium did not contain glutamate, this was likely the result of increased bioconversion of glutamine. However, for higher initial arginine concentrations, GABA accumulation was markedly decreased to levels similar to those of control cultures. This was probably caused by excessive medium alkalinization and consequent inhibition of glutamate decarboxylation system.

Arginine at 29 mM (5 g L^-1^) was chosen for further experiments in fermenter, since this culture condition was characterized by the highest specific GABA production (i.e., amount of GABA/final biomass ratio). Such arginine supplementation did not significantly increase the μ_max_ but doubled final biomass with respect to control conditions (**Table 1**). Although arginine was completely exhausted at the growth arrest at 6 h, the extracellular pH at this point, was not much more alkaline than in control conditions. However, in arginine-supplemented cultures a twofold higher glucose amount was consumed leading to a final lactate concentration of about 123.3 mM which likely had a neutralizing effect on the NH_3_ released via the ADI pathway (**Table 1**). Consistently, ornithine and citrulline accumulated in the culture broth and levels were proportional to arginine consumption. No putrescine was detected.

As observed in tube-cultures, arginine supplementation also enhanced GABA production. Accumulation of increased amounts of GABA cannot be explained only by the higher biomass achieved in arginine-supplemented cultures, since biomass was increased by twofold whereas GABA accumulation was enhanced by fourfold at 48 h. On the other hand, specific glutamine consumption rate was similar to values measured in control conditions. These observations suggest that arginine directs a higher proportion of glutamine metabolic flux towards GABA production diverting it from other pathways.

#### Effect of Malate

Cultures of *L. lactis* NCDO 2118 in tubes containing glucose-CDM medium supplemented with malate concentration ranging from 0 to 50 g L^-1^ (0–373 mM) were performed (**Figure 3C**). With increasing malate supplementation, final pH and μ_max_ were affected in somehow similar ways as what observed in arginine fortified cultures, i.e., final pH progressively raised, while μ_max_ was lower for higher malate supplementation. Taking into account that supplemented concentrations of malate were more than twofold higher than for arginine, malate supplementation had more limited effects on acid neutralization (final pH with 50 g L^-1^ malate supplementation was about 7). On the contrary, such high malate concentration negatively affected μ_max_ (i.e., it was reduced from 1 to 0.4 h^-1^). A moderate final biomass increase (up to 1.4 g L^-1^) was observed up to malate concentration of 40 g L^-1^ (298 mM). Higher malate supplementation caused reduction of final biomass to levels lower than control conditions.

Cultures of *L. lactis* NCDO 2118 in glucose-CDM medium containing 20 g L^-1^ (149 mM) malate were performed in fermenter also. This condition was chosen since the GABA produced was significantly increased compared to the reference condition while the growth rate was similar. Maximal specific growth rate, final biomass and final extracellular pH were coherent with results obtained in tube cultures with the same malate concentration. This condition was the one stimulating the consumption of the highest amount of glucose compared to reference conditions or supplementations with glutamate or arginine at 48 h (**Table 1**). Actually, glucose was almost depleted after 12 h of culture, although specific glucose consumption rate was similar the other growth conditions tested. However, malate supplementation did not stimulate any further GABA accumulation with respect to control conditions (**Table 1**). Curiously, malate seems to increase arginine consumption rate (2.69 mmol g^-1^ h^-1^) with respect to cultures on CDM.

### Cultures Supplemented with Glutamate Plus Arginine or Glutamate Plus Malate

#### Effect of Simultaneous Arginine and Glutamate Supplementation

The simultaneous addition of arginine and glutamate to the glucose-CDM medium was studied in fermenter. The concentrations of arginine (5 g L^-1^, 29 mM) and glutamate (5 g L^-1^, 34 mM) were chosen according to the individual fermenter conditions previously tested. A slightly decreased maximal growth rate (μ_max_ = 0.89 h^-1^) was observed while final pH was similar to cultures supplemented with glutamate only (**Table 1**). Final biomass at the growth arrest was also significantly higher like cultures supplemented with arginine only. However, GABA production was strongly enhanced since 8.6 mM were accumulated in the medium, that is almost threefold higher that in cultures supplemented with glutamate only. This was the highest amount of GABA accumulation observed in this study and was likely the result of both higher substrate availability (i.e., glutamate supplementation) and stimulation of GABA production by arginine, confirming results obtained in cultures supplemented with arginine only.

#### Effect of Simultaneous Malate and Glutamate Supplementation

In a similar way, the effect of simultaneous malate and glutamate supplementation was tested in fermenter. This condition noticeably reduced μ_max_ (0.81 h^-1^) with respect to control conditions as observed in cultures supplemented with glutamate only (**Table 1**). However, the specific glucose consumption rate was similar (**Table 1**). Final biomass and extracellular pH at the growth arrest, as well as glucose consumption and lactate production at 48 h were higher than the reference but similar to that measured in cultures fortified with malate only. Hence, contribution of glutamate to energy metabolism seems negligible. This is confirmed by the fact that in this condition only 2.54 mM of GABA was accumulated at 48 h. This value is not significantly different from GABA amounts observed in culture supplemented with glutamate only. These data, taken together with the fact that in malate-plus–glutamate-supplemented cultures biomass production was higher than in culture supplemented with glutamate only, suggest that malate somehow repressed GABA production pathway(s). On the other hand, specific malate consumption rate was slightly lower than in cultures supplemented with malate only (**Table 1**).

### Cultures Supplemented with Glutamate Plus Arginine Plus Malate

Cultures of *L. lactis* NCDO 2118 were performed in CDM supplemented with 5 g L^-1^ arginine (29 mM), 20 g L^-1^ malate (149 mM) and 5 g L^-1^ glutamate (34 mM). Since usual glucose concentration (20 g L^-1^, 110 mM) was exhausted after 10 h of growth (in agreement with the high sugar consumption rate observed in other malate-supplemented cultures) initial glucose concentration was increased to 45 g L^-1^ (250 mM) so as to avoid growth limitation by sugar depletion.

As for other malate supplemented cultures, growth was at slightly lower μ_max_ (0.85 h^-1^) with respect to control cultures supplemented with glutamate. Simultaneous supplementation of glutamate, malate, and arginine had additive effects on: (i) attenuating extracellular acidity caused by lactic acid accumulation (353.3 mM was produced 48 h after inoculum), since pH at both time of growth arrest and after 48 h was the highest observed among the growth conditions tested in this study and (ii) increasing biomass formation up to 2.2 g L^-1^. Growth arrest occurred 9 h after inoculum. At that time, while malate had already been depleted, only 71.1 mM of glucose had been consumed, leading to accumulation of 228.05 mM lactic acid. The high amount of lactic acid observed in these conditions could contribute to growth arrest for reasons independent from acidification.

The specific consumption rates for glucose and glutamine were of the same order of magnitude of values measured in control conditions (**Table 1**). Specific malate consumption rate was slightly lower than in the other malate-supplemented cultures described above, while arginine was consumed at a highest specific rate (14.58 mmol g^-1^ h^-1^, **Table 1**).

**FIGURE 5:**
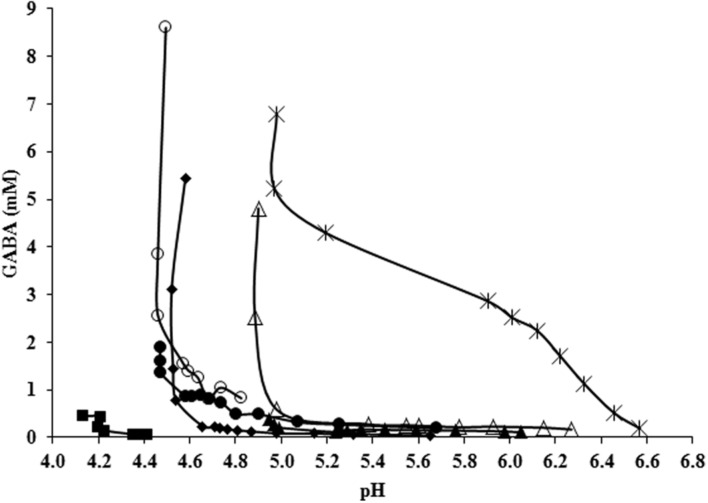
**GABA production (mM) as a function of the pH of the medium in all conditions tested at 48 h of culture: CDM (■), CDM with glutamate (◆), with arginine (●), with malate (▲), with arginine and glutamate (○), with malate and glutamate (△), and with arginine, malate, and glutamate (★)**.

Finally, GABA was produced until a maximal concentration of 5.22 mM at 48 h, which is higher than amounts accumulated in glutamate- and glutamate-plus–malate-supplemented cultures but lower than those accumulated in glutamate-plus–arginine supplemented cultures. This data confirmed that arginine enhances while malate decreases GABA accumulation. Interestingly, simultaneous glutamate–malate–arginine supple- mentation triggered an earlier production of GABA with respect to all the other tested conditions: a concentration of 2.86 mM was already detected 12 h after inoculum and it reached 4.28 mM at 24 h. This earlier production of GABA can be observed in **Figure 5** which combines the kinetics of the different experiments. When glutamate, arginine and malate were contemporarily supplemented to *L. lactis* NCDO 2118 cultures, GABA production started at the beginning of the exponential growth phase and at a pH as high as 6.6 while only tardive GABA production in acidic conditions (pH < 5.1) was observed in all other tested conditions.

## Discussion

Several systems can be activated or enhanced by LAB to attenuate acidic environments and/or to improve metabolic energy. If the most obvious strategy involves *F*_0_*F*_1_-ATPase, other mechanisms such as the ADI pathway or the decarboxylation of malate and amino acids can be used to neutralize or reduce acidity (Budin-Verneuil et al., 2004). ADI pathway and malate decarboxylation by MLF are often present in LAB which live in wine ecological niche in which both malate and arginine are abundant. In LAB, biogenic amine production (including GABA biosynthesis) through amino acid decarboxylation usually occurs in response to adverse conditions, e.g., as a mean to counteract acidic environments (Van de Guchte et al., 2002) and to obtain metabolic energy when the primary substrates (e.g., glucose) are exhausted (Molenaar et al., 1993; Pessione et al., 2010). However, little is known about the relative role of the considered metabolic pathways in pH homeostasis, their possible synergistic/antagonist effects and consequences on global metabolism and growth of LAB. In the present study, the contribution of energy supplying/alkalinizing routes (i.e., ADI pathway, malate fermentation and production of GABA), to growth and metabolism of *L. lactis* NCDO 2118 and their reciprocal relationships was investigated. Effects of supplementation of different amounts of substrates (arginine, malate, and glutamate) or their mixtures on growth (specific growth rate, final biomass), substrate consumption and GABA production have been quantitatively determined.

### Growth Parameters

In all tested conditions, the growth profile was characteristic of *L. lactis* cultures, in which the growth rate was maximal in the early phase and then progressively decreased. Growth arrest was likely caused by low pH and/or lactic acid accumulation in every condition considered here. The increase in final biomass seems inversely correlated to the pH decrease: the higher the initial concentration of arginine or malate, the higher was likely the activation of corresponding acid resistance mechanisms (ADI pathway and MLF, respectively) resulting in weaker and slower pH decrease, hence the growth could be supported for longer time periods. It is worth noting that while in arginine-supplemented cultures the pH acidification caused by lactic acid accumulation by glycolysis is neutralized by release of ammonia, pH increase by MLF leads to additional lactate production. It can be speculated that huge lactic acid accumulation is likely the main growth inhibiting factor in malate-supplemented cultures, for reasons independent from medium acidification. For instance, this could explain why in cultures supplemented with 149 mM malate cell growth stopped at lower final biomass, i.e., 1.86 g L^-1^, and higher pH, i.e., pH = 5.76, with respect to cultures supplemented with arginine. However, we cannot exclude that arginine and/or malate stimulate biomass production by additional mechanisms (other than homeostasis), such as production of biosynthetic intermediates, for instance through malate conversion by malic enzyme (ME; Landete et al., 2013) or arginine conversion to pyrimidine precursors.

The maximal glucose consumption rate was similar in all tested conditions, and the central metabolism remained homolactic. From this macro-kinetic analysis, no evidence that glycolytic flux was affected by alternative acid-resistance related pathways could be inferred. This is in agreement with the observation that the expression of two enzymes of the central metabolism, namely phosphoglucomutase and pyruvate dehydrogenase, was unaffected by arginine or malate in *L. hilgardii* (Lamberti et al., 2011). The efficiency of arginine, glutamate, and malate dissimilation pathways of *L. lactis* NCDO 2118 was very diverse, as demonstrated by specific substrate consumption rates. Malate was consumed at the highest rate (maximal *q*_malate_ was comprised between 21.5 and of 29.5 mmol g^-1^ h^-1^), followed by the arginine (maximal *q*_Arg_ comprised between 1.2 and of 14.6 mmol g^-1^ h^-1^), and finally by glutamate (maximal *q*_Glu__≤_ 0.2 mmol g^-1^ h^-1^). These data suggest that MLF is the most rapid system for neutralizing acidity in this strain. In fact, the slowest acidification was observed in cultures supplemented with malate. Malate consumption rate seems negatively affected by both glutamate and arginine supplementation, suggesting that in these conditions MLF is inhibited. It has been previously reported that malate consumption was not affected by histidine in the culture medium in *L. hilgardii* ISE5211 (Mazzoli et al., 2009; Lamberti et al., 2011). Similarly, ornithine–putrescine conversion does not affect MLF in *Oenococcus oeni* (Mangani et al., 2005). Hence amino acid and malate decarboxylation can be activated in parallel without reciprocal interference in these two wine-isolated strains. This is not the case in *L. lactis* NCDO 2118 in which also glutamate decarboxylation is negatively affected by malate suggesting that these routes are competing. One possible explanation for this different behavior is that *L. lactis* NCDO 2118 has been isolated from another ecological niche (frozen peas) and can be encountered in milk fermentation. Arginine consumption rate is strongly influenced by environmental conditions. Notably, both arginine and malate supplementation increases maximal specific consumption rate of arginine (**Table 1**). These data suggest that both arginine and malate may activate ADI pathway. The activation of ADI by arginine has been previously described for many LAB species (Manca de Nadra et al., 1988; Tonon et al., 2001; De Angelis et al., 2002; Lamberti et al., 2011). However, positive regulation of ADI by malate has never been reported so far. Proteomic analyses showed that the expression of ADI pathway enzymes is not affected by malate in *L. hilgardii* (Lamberti et al., 2011). Furthermore, malate inhibited arginine consumption in some lactobacilli and pediococci isolated from wine (Araque et al., 2011). However, Rallu et al. (1996) previously suggested that lactic acid can activate arginine metabolism, including ADI pathway, in *L. lactis*. We can hypothesize that malate is able to enhance ADI pathway in *L. lactis* NCDO 2118 through additional lactate production by MLF although a direct activation of this pathway by malate cannot be excluded to be specific to the strain considered in this study.

### GABA Production

γ-aminobutyric acid production was clearly dependent on the environmental conditions. In absence of glutamate, only low amounts of GABA were produced likely as a consequence of the conversion of glutamine (which is a CDM component) into glutamate. The higher glutamate supplementation, the higher the final concentration of GABA (**Figure 3A**). However, relationship between the amount of supplemented glutamate and produced GABA is not linear but rather looks like a Michaelis–Menten plot. Since glutamate amount did not affect significantly final biomass and growth rate, these data suggest that the same amount of enzymes involved in glutamate/GABA conversion (i.e., GAD and/or glutamate/GABA antiporters) were present in all these conditions, i.e., glutamate did not improve their biosynthesis. In these conditions, the system is semi-saturated for glutamate concentrations of about 7 mM. The present data therefore confirm previous results obtained on *L. lactis* NCDO2118 which indicated that glutamate supplementation did not induce overexpression of GAD (Mazzoli et al., 2010).

Arginine supplementation significantly enhanced GABA production in both absence or presence of glutamate fortification. The latter condition corresponds with the production of the highest GABA amount observed in this study (8.6 mM). It is possible to hypothesize that arginine can replace glutamine/glutamate in some metabolic function, allowing a higher proportion of these compounds to be directed towards GABA production. For instance, it is well known that in *L. lactis* glutamine can be converted to carbamoyl phosphate, the building block for pyrimidine biosynthesis, by carbamoyl phosphate synthase (EC 6.3.5.5) (Martinussen and Hammer, 1998). Since also arginine can generate carbamoyl phosphate through the ADI pathway, it could replace glutamine allowing it to be used for GABA biosynthesis. Similarly, arginine could replace glutamate as amino group donor allowing higher glutamate conversion to GABA.

An analysis of GABA production as a function of the pH of the culture confirmed the strong dependency of this metabolic system on pH (**Figure 5**). Significant GABA production was observed only at pH lower than 5.1 during stationary phase (**Figure 5**), thus confirming previous observations on the same strain (Mazzoli et al., 2010). These data also agree with recent studies reporting that acidic pH is necessary for activating glutamate/GABA antiport by GadC (Lu et al., 2013; Tsai et al., 2013). However, when glutamate, arginine and malate were contemporarily supplemented to *L. lactis* NCDO 2118 cultures, GABA production started at the beginning of the exponential growth phase and at a pH as high as 6.6 (**Figure 5**). At pH 6.6, the intracellular pH is estimated to be close to the neutrality (Even et al., 2002) and is not compatible with the GAD activity that was found to be highly inhibited above pH 5.4 in our bacteria (results not shown). This suggests that another enzyme probably ensures decarboxylation of glutamate in neutral conditions. As far as we know, this is the first evidence of significant GABA production during the exponential growth phase and at nearly neutral pH. The biomass profile could not explain this production since it was similar to cultures supplemented with arginine plus glutamate, where early activation did not occur. Since this effect was not observed when each of these three metabolites was supplemented individually or as binary mixtures (glutamate plus arginine or glutamate plus malate) it can be speculated that the simultaneous activation of ADI pathway and MLF is susceptible to activate GABA production, even at higher pH and during exponential growth. Although more work is needed to understand the biochemical basis of this phenomenon, this observation could have significant impact on industrial GABA production process.

## Conclusion

Acidic environments constitute a major stress for LAB which developed several acid-counteracting systems which include ADI pathway, MLF, and amino acid decarboxylation. Regulation and reciprocal interactions of these pathways seem to vary among microbial species. The present study indicated that glutamate decarboxylation plays minor roles in *L. lactis* NCDO 2118 physiology with respect to malate and arginine dissimilation. In fact, glutamate supplementation had very limited effect in neutralizing acidity and in stimulating biomass production in contrast to results obtained through malate and arginine supplementation. Furthermore, GABA production was restrained to narrower environmental conditions than malate or arginine dissimilation, since both acidic pH (pH < 5.1) and stationary phase were generally required for its activation. However, this study indicated some strategies which enabled activation of glutamate decarboxylation system outside of this compass. Notably, arginine was able to strongly stimulate GABA production, while simultaneous addition of arginine and malate was able to trigger glutamate decarboxylation in earlier growth phase (i.e., exponential phase) at near neutral pH. Even if understanding the molecular basis of these phenomena will require further studies, these results are valuable tracks for developing more performant industrial processes for enhanced and earlier GABA production through fermentation.

## Author Contributions

VL: Contribution to acquisition, analysis and interpretation of the data, contribution in drafting the article. CY and WN: contribution to experimental and chemical analysis. RM, EP, and MC-B: contribution to interpretation of data and in drafting the article. PL: design of the work, analysis and interpretation of data, writing of the manuscript.

## Conflict of Interest Statement

The authors declare that the research was conducted in the absence of any commercial or financial relationships that could be construed as a potential conflict of interest.
